# A nationwide survey concerning practices in pessary use for pelvic organ prolapse in The Netherlands: identifying needs for further research

**DOI:** 10.1007/s00192-015-2697-6

**Published:** 2015-06-11

**Authors:** Joost Velzel, Jan Paul Roovers, C H Van der Vaart, Bart Broekman, Astrid Vollebregt, Robert Hakvoort

**Affiliations:** Department of Obstetrics and Gynecology, Academic Medical Center Amsterdam, Meibergdreef 9, 1105 AZ Amsterdam, The Netherlands; Department of Obstetrics and Gynecology, Spaarne Ziekenhuis, Hoofddorp, The Netherlands; Department of Obstetrics and Gynecology, University Medical Centre, Utrecht, The Netherlands; Department of Obstetrics and Gynecology, Sint Franciscus Gasthuis, Rotterdam, The Netherlands

**Keywords:** Prolapse, Pessary, Survey, Prolapse management

## Abstract

**Introduction and hypothesis:**

To identify practice variation in management of patients with a vaginal pessary for pelvic organ prolapse (POP).

**Methods:**

A nationwide survey was sent to all Dutch gynecologists with a special interest in urogynecology.

**Results:**

The response rate was 59 %. Of the respondents, 13 % had a written protocol for pessary treatment in their department. Pessary treatment was proposed by 69 % of respondents as a treatment option. Counseling about side effects varied. All respondents provided information concerning the possibility of serious vaginal discharge. Concerning this side effect, 15 % of the respondents stated that it occurs in 5 – 20 % of patients, 27 % that it occurs in 20 – 40 % of patients, and 57 % that it occurs in more than 40 % of patients. Another item concerned counseling for the likelihood of vaginal blood loss. All respondents provided information concerning the possibility of vaginal blood loss. Concerning this side effect, 53 % of the respondents stated that it occurs in 5 – 20 % of patients, 33 % that it occurs in 20 – 40 %, and 14 % that it occurs in more than 40 % of patients. Follow-up after initial placement was done by 69 % of the respondents at 2 – 6 weeks, by 2 % at 8 weeks, and by 29 % at 12 weeks or more. Most (98 %) of the respondents extended the interval between visits when the patient had no complaints, and 96 % of the respondents reported that they routinely instruct patients about self-management.

**Conclusions:**

Pessaries are suggested as a treatment option by a majority of gynecologists, but practice varies widely. We consider that the variation in practice is due to a lack of available protocols and lack of evidence.

## Introduction

Pelvic organ prolapse (POP) is a common condition in adult women [1]. For the relief of symptoms related to POP conservative treatment options include life-style advice, pelvic physiotherapy and pessary treatment [2–4]. The aim of pessary treatment is to restore the anatomy of the visceral organs of the pelvic cavity by inserting a device into the vagina. The reported subjective cure rates vary between 60 % and 80 % [5, 6]. Many patients prefer this treatment over surgery, as it is unlikely to cause serious morbidity and normal activities can be continued [7–10]. Successful pessary fitting and long-term success have been documented in up to 75 % of women with symptomatic POP [11–14]. The reason for the high proportion (80 – 85 %) of gynecologists placing pessaries may be the ease with which they can be inserted and removed, their efficacy and the low complication rate [15, 16].

Although large groups of women receive these devices, a national guideline regarding POP was not available in The Netherlands at the time of our survey. The proportion of patients made aware of this treatment option, which patients are most eligible for this treatment, and how long follow-up intervals should be were unknown. Ignorance concerning these issues may result in practice variation and suboptimal treatment of patients. Therefore, the aim of the present survey was to identify current practice and variation in practice concerning treatment with vaginal pessaries for POP.

## Materials and methods

An invitation to participate was sent to all 151 members of the Dutch working party for urogynecology of the Dutch College for Obstetrics and Gynecology. This membership represents 15.4 % of the 981 Dutch gynecologists. We expected all members of the Dutch working party for urogynecology to be familiar with the indications for and performance of pessary treatment. Participants were asked to answer the survey using an online system (SurveyMonkey®). A reminder was sent to those who had not responded 4 weeks after the first request. Nonresponders were sent a third request 4 weeks after the second request.

The questionnaire was developed specifically for the survey based on previous questionnaires [15, 16] and by a panel of four experts in urogynecology. The questionnaire included both multiple choice and open questions addressing the following topics: characteristics of gynecologists and hospitals, selection of patients, follow-up management and counseling patients about side effects, effectiveness and the option of self-management. We asked the respondents to answer as if considering a patient without a previous pessary fitting trial. The full questionnaire can be found in Table 5 in the Appendix. Ethics review board approval was not applied for.

Data from the completed questionnaires were used to calculate frequencies and percentages of respondents answering per question per topic. Descriptive data analysis was performed using SPSS 19.0 (SPSS Statistics UK, SPSS Inc., Chicago, IL).

## Results

After three requests, a total of 91 of 151 gynecologists (59 %) in 63 of 80 hospitals (79 %) had responded. In 98 % of the responding hospitals a gynecologist with a special interest in urogynecology was employed. A written protocol for indication, insertion and follow-up of pessary treatment was available in 13 % (Table 1).

**Table 1:** Characteristics of the hospitals of responding gynecologists

		No. (%) of respondents
Type of hospital	General hospital	26 (29)
	Teaching hospital	55 (60)
	Academic hospital	9 (10)
	Private practice	1 (1)
New patients with vaginal prolapse annually	0 – 400	24 (26)
	401 – 800	46 (51)
	>800	21 (23)
Vaginal prolapse surgery procedures annually	0 – 100	14 (15)
	101 – 200	52 (57)
	>200	25 (28)
Gynecologist with special interest urogynecology	Yes	89 (98)
	No	2 (2)
Existence of written protocol for pessary use	Yes	12 (13)
	No (but consensus among caregivers)	54 (59)
	No	25 (27)

Pessary placement for the treatment of POP was proposed by 69 % of respondents always, by 17 % sometimes, and by 14 % never. Prolapse of the anterior compartment and apical compartment were considered the most suitable indications for pessary treatment (99 % and 96 % of the respondents, respectively). Concerning the decision to start initial treatment with a pessary, 62 % of the respondents stated that they were not influenced by the stage of prolapse, and 36 % stated that patient age was a decisive factor, with younger patients being less likely to receive a pessary (Table 2).

**Table 2:** Selection of patients

		No. (%) of respondents
Standard information about the option of a pessary	Yes	63 (69)
	Occasionally	15 (17)
	No	13 (14)
Type of prolapse thought to be most suitable for pessary^a^	Prolapse anterior compartment	88 (99)
	Prolapse middle compartment	85 (96)
	Prolapse posterior compartment	9 (11)
	Stress incontinence	11 (12)
	Urge incontinence	8 (9)
	Constipation	0 (0)
	Obstructed defecation	26 (29)
Influence of prolapse stage on decision	Yes	35 (38)
	No	56 (62)
Influence of patient age on decision	Yes	33 (36)
	No	58 (64)

^a^More answers possible

Table 3 shows the protocols and practices of gynecologists concerning follow-up after initial placement. The interval between initial placement and first follow-up varied from 2 weeks to 16 weeks. The first follow-up visits were at 2 weeks (17 % of respondents), 3 weeks (15 %), 4 weeks (14 %), 6 weeks (23 %), 8 weeks (2 %), 12 weeks (23 %), and 16 weeks (6 %).

**Table 3:** Follow up

		No. (%) of respondents
First follow-up visit (weeks)	2	15 (17)
	3	14 (15)
	4	13 (14)
	6	21 (23)
	8	2 (2)
	12	21 (23)
	16	5 (6)
Professional responsible for first follow-up visit	Same caregiver	30 (33)
	Same caregiver, later general practitioner	48 (53)
	Same caregiver or general practitioner	1 (1)
	Specialist nurse	1 (1)
	Patient wishes	11 (12)
Timing of follow-up visits after initial placement^a^	Same interval continued	15 (16)
	Shorter intervals if complaints	27 (30)
	Longer intervals if no complaints	90 (99)
Prescription of estrogens (oral and vaginal)	Yes	12 (13)
	When indicated (vaginal atrophy)	66 (73)
	When indicated (other than atrophy)	8 (9)
	No	5 (5)

^a^More answers possible

Regarding the first follow up visit, 78 respondents (86 %) stated that it was carried out by a gynecologist, 2 % delegated this care to either the general practitioner (GP) or a nurse practitioner, and 12 % stated that the patient could choose between the above professionals. If there were no patient complaints, 99 % of respondents increased the interval between visits. Estrogens were prescribed routinely by 13 % of respondents, only when vaginal atrophy was present by 73 %, and when indications other than atrophy, for example irritation, were present by 9 %; 5 % of respondents never prescribed estrogens.

Counseling about side effects varied. All respondents provided information concerning the possibility of serious vaginal discharge. Concerning this side effect, 15 % of the respondents stated that it occurs in 5 – 20 % of patients, 27 % that it occurs in 20 – 40 % of patients, and 57 % that it occurs in more than 40 % of patients. Another item concerned counseling for the likelihood of vaginal blood loss. All respondents provided information concerning the possibility of vaginal blood loss. Concerning this side effect, 53 % of the respondents stated that it occurs in 5 – 20 % of patients, 33 % that it occurs in 20 – 40 %, and 14 % that it occurs in more than 40 % of patients. Concerning the success rates of pessary treatment, 5 % of respondents informed patients that pessary treatment is successful in only 5 – 20 % of patients, 46 % that it is successful in 20 – 50 % of patients, and 48 % that it is successful in more than 50 % of patients. The likelihood of eventually receiving surgical treatment following failure of pessary treatment was stated to be 0 – 25 % by 24 % of respondents, 25 – 50 % by 56 % and more than 50 % by 20 %. Of the respondents who proposed pessaries as a treatment option, 96 % informed the patient about the possibility of self-management (Table 4).

**Table 4:** Patient information and self-management

			No. (%) of respondents
Likelihood of side effects of pessary use	Vaginal discharge	5 – 20 %	14 (15)
		20 – 40 %	25 (27)
		>40 %	52 (57)
	Vaginal blood loss	5 – 20 %	48 (53)
		20 – 40 %	30 (33)
		>40 %	13 (14)
Likelihood of surgical treatment for POP after pessary treatment	0 – 25 %		22 (24)
	25 – 50 %		51 (56)
	50 – 75 %		17 (19)
	75 – 100 %		1 (1)
Likelihood of pessary extrusion	5 – 15 %		36 (40)
	15 – 30 %		45 (49)
	30 – 50 %		8 (9)
	>50 %		2 (2)
Likelihood of that pessary treatment will be effective/satisfactory	5 – 20 %		5 (5)
	20 – 50 %		42 (46)
	>50 %		44 (48)
Gynecologist giving advice about self-management	Always		46 (51)
	Regularly		41 (45)
	No		4 (4)
Gynecologists giving instructions about self-management	Yes, always		48 (53)
	Regularly		39 (43)
	No		4 (4)
Patients successful in self-management returning to outpatient clinic	Yes		36 (40)
	No		55 (60)

## Discussion

A nationwide survey was performed to quantify the variation among gynecologists in the practice of pessary treatment in women with symptomatic POP. A low percentage (only 13 %) of respondents stated that they had a written protocol available in their department. A relatively high percentage (69 %) of respondents routinely suggested pessary treatment for POP. A considerable variation was found in counseling about vaginal discharge and vaginal blood loss as possible side effects. Furthermore, the intervals between placement and the first follow-up visit varied greatly. A majority of the respondents reported that they routinely instruct the patient about self-management.

 Before discussing these results in more detail, we address some limitations of this study. A criticism might be that the 59 % response rate did not reflect overall clinical practice. However, the respondents represented 79 % of Dutch departments. Failure of a proportion of potential participants to respond means that trends that could have been recognized were missed. There could have been a reporting bias favoring younger potential participants due to the online survey tool used. However, it is not clear how this could have affected the results. As previous surveys on this topic had response rates of 21 % [15] and 55 % [16] we consider that the response rate can be regarded as high. Another possible limitation was the way in which questions were formulated. To be able to obtain answers that could be more easily analyzed multiple choice questions were mainly used, which may not always have reflected actual practice. Unfortunately, this is inherent in the survey format.

Also GPs contribute to this type of care, estimated in The Netherlands to be around 20 % of the total amount of care, but this group was not interviewed. There is no expertise in pessary treatment for POP among other groups in The Netherlands, for example nurse practitioners. It would have been interesting to perform a similar survey among GPs to gain an insight into their use of pessaries in the management of POP. Because of the relatively small contribution of GPs to this care we decided not to include GPs in the study. Furthermore, we recognize that this survey only dealt with daily practice in The Netherlands. Similar surveys in more countries could generate information of more clinical value.

The study clearly demonstrated that a considerable proportion (69 %) of gynecologists provide informs about the possibility of pessary treatment. The available literature clearly indicates that a high proportion of patients become long-term users with a high patient satisfaction [10, 13, 17]. This may be a result of the high percentage of patients who receive information about this treatment. In general, respondents considered pessary treatment for prolapse of the anterior and apical compartments as more successful than pessary treatment for prolapse of the posterior compartment. In literature, there is no definitive evidence that pessary use for posterior compartment prolapse is not as successful as pessary treatment for anterior wall prolapse [12, 18, 19]. The two largest studies have demonstrated [12, 18] no difference in the success of pessary treatment in relation to the type and severity of prolapse, and conclude that patients should not be selected for pessary treatment on the basis of the type of prolapse.

Information given to patients about adverse effects of pessary treatment varied to a great extent and the information that was given about the occurrence of side effects was often not in line with existing evidence. Vaginal discharge has been reported to occur in 25 % of patients [20] and vaginal blood loss in 6 – 46 % of patients [20–22] at 1 year after treatment. A large proportion (57 %) of respondents considered (and possibly discussed with patients) that serious vaginal discharge occurs more frequently than it actually does. These discrepancies between perceived and actual complication rates may negatively affect the willingness of the gynecologist to propose and of the patient to undergo pessary treatment.

Large differences in follow-up intervals were reported by the respondents. Shorter intervals could be associated with unnecessary higher costs per patient. However, there is no clarity in the literature as to the ideal or minimal follow-up intervals after initial placement. Neither is there any evidence concerning the proportion of patients who are able to learn to clean and replace the pessary themselves. A majority of the respondents reported that they routinely instruct patients about self-management, including how to change and clean the pessary at home. However, from this survey we cannot determine the success of this advice and training.

## Conclusions

This survey clearly showed that there is large variation in the use of pessaries in the management of POP. This includes the information patients are provided with. Other variations concern follow-up after placement. A prospective study regarding effectiveness and consequences of the use of pessaries in the management of POP, and patient satisfaction with this approach, is needed.

## Appendix 1

**Table 5:** Full survey questionnaire

Topic 1: Characteristics of gynecologists and hospitals		
A] In what type of department do you work?	General hospital	
	Teaching hospital	
	Academic hospital	
	Private practice	
B] How many new patients with vaginal prolapse are seen in your clinic annually?	0 – 200	
	201 – 400	
	401 – 600	
	601 – 800	
	>800	
C] How many vaginal prolapse surgery cases are there in your clinic annually?	0 – 100	
	101 – 150	
	151 – 200	
	201 – 300	
	>300	
D] How many new patients with incontinence are seen in your clinic annually?	0 – 25	
	26 – 50	
	51 – 75	
	76 – 100	
	>100	
E] Is there a gynecologist with a special interest in urogynecology employed in your clinic?	Yes	
	No	
F] Is there a written protocol for pessary use in your clinic?	Yes	
	No, but consensus between caregivers	
	No	
Topic 2: Selection of patients		
A] Do you propose pessary placement as your standard initial treatment?	Yes	
	Not in some cases	
	No	
B] What type of prolapse or complaint is most suitable for pessary treatment (multiple-choice question)?	Prolapse anterior compartment	
	Prolapse middle compartment	
	Prolapse posterior compartment	
	Stress incontinence	
	Urge incontinence	
	Constipation	
	Obstructed defecation	
C] Does stage of prolapse influence pessary treatment?	Yes	
	No	
D] Does patient age influence pessary treatment?	Yes	
	No	
Topic 3: Follow-up management		
A] What is the interval to the first follow-up after initial placement in weeks?	2 weeks	
	3 weeks	
	4 weeks	
	6 weeks	
	8 weeks	
	12 weeks	
	16 weeks	
B] Which professional is responsible for the first follow-up visit after initial placement	Same caregiver	
	Same caregiver, later on general practitioner	
	Same caregiver or general practitioner	
	A specialist nurse	
	Patient wishes	
C] Do the intervals between follow-up visits change after initial placement (multiple-choice question)?	Same interval continued	
	Shorter intervals if complaints	
	Longer intervals if no complaints	
D] Do you prescribe estrogens (oral or vaginal use)?	Yes	
	When indicated (vaginal atrophy)	
	When indicated (other than atrophy)	
	No	
Topic 4: Information gynecologists provides to patients including the option of self-management		
A] How often does vaginal discharge occur due to pessary treatment for prolapse?	5 – 20 %	
	20 – 40 %	
	40 – 60 %	
	60 – 75 %	
	>75 %	
B] How often does vaginal blood loss occur due to pessary treatment for prolapse?	5 – 20 %	
	20 – 40 %	
	40 – 60 %	
	60 – 75 %	
	>75 %	
C] What is the average chance on getting surgical treatment for prolapse after pessary treatment?	0 – 25 %	
	25 – 50 %	
	50 – 75 %	
	75 – 100 %	
D] How often does pessary extrusion occur?	5 – 15 %	
	15 – 30 %	
	30 – 50 %	
	>50 %	
E] What Information do you give about the chance that pessary treatment will be effective/satisfactory?	5 – 20 %	
	20 – 50 %	
	>50 %	
F] Do you give advice on self-management?	Always	
	Regularly	
	No	
G] Do you give instructions on self-management?	Always	
	Regularly	
	No	
